# Ethnic and Gender Differences in 10-Year Coronary Heart Disease Risk: a Cross-Sectional Study in Hawai‘i

**DOI:** 10.1007/s40615-020-00851-2

**Published:** 2020-08-31

**Authors:** Claire Townsend Ing, Hyeong Jun Ahn, Rachel Kawakami, Andrew Grandinetti, Todd B. Seto, Joseph Keawe’aimoku Kaholokula

**Affiliations:** 1 Department of Native Hawaiian Health, University of Hawai‘i, Honolulu, HI USA; 2 Department of Quantitative Health Sciences, University of Hawai‘i, Honolulu, HI USA; 3 Grand Canyon University, Phoenix, AZ USA; 4 Office of Public Health Studies, University of Hawai‘i, Honolulu, HI USA; 5 Department of Medicine, University of Hawai‘i and Queen’s Medical Center, Honolulu, HI USA

**Keywords:** Ethnic differences, Cardiovascular disease risk, Native Hawaiian, Filipino, Japanese, Non-Hispanic White

## Abstract

**Background:**

Cardiovascular disease (CVD) is the leading cause of death in the US. In Hawai‘i, Filipinos and Native Hawaiians have the highest rates of CVD-related risk factors. CVD risk across these ethnic groups has not been examined. This cross-sectional study examines 10-year CVD risk as determined by the Framingham Risk Score (FRS) across ethnic groups in Hawai‘i, controlling for clinical, demographic, and psychosocial factors.

**Methods:**

This study includes secondary data analysis of the Kohala Health Research Project dataset. All non-pregnant adults (≥ 18 years of age) who resided in the community of interest during the study period were eligible to participate with 1462 participants completing the clinical examination and surveys. This analysis included clinical, demographic, and psychosocial variables. Ethnic differences were examined using the chi-squared test and one-way ANOVA. Multiple linear regression on FRS was conducted and least square means of FRS were calculated.

**Results:**

Data from 1146 individuals were analyzed. Participants were 44.4% Native Hawaiian, 15.4% Filipino, 15.3% Japanese, and 25% non-Hispanic White; 55.4% were female and had a mean age of 48.8 years. For males, the unadjusted Japanese mean FRS was significantly higher compared with the other ethnic groups. For females, Filipino and Japanese mean FRS were significantly higher compared with Native Hawaiians and non-Hispanic Whites. In the fully adjusted model, there were no ethnic group differences in FRS among males and Filipinos had significantly higher FRS compared with non-Hispanic White among females.

**Conclusions:**

This cross-sectional community-based epidemiological study examined ethnic differences in CVD risk after adjusting for age, depression, social support, and acculturation. The results suggest that some ethnic differences in CVD risk persist even after controlling for confounders but that recalibration of risk assessment is necessary.

## Background

Cardiovascular disease (CVD) is the leading cause of death in the United States (US), accounting for more deaths than homicide, suicide, automobile accidents, HIV/AIDS, alcohol use, and drug use combined [1]. The current total cost, including medical and indirect costs, of CVD conditions (i.e., hypertension, coronary heart disease, coronary heart failure, stroke, and other heart diseases) is $555 billion, which is projected to more than double by 2035 [2]. Addressing CVD is a major public health, economic, and research priority [2, 3]. Biologic risk factors for CVD, including hypertension, obesity, and cholesterol, are well documented. For instance, hypertension causes 50% of ischemic strokes, elevates risk for hemorrhagic stroke and coronary heart disease, and accounts for almost half of CVD deaths [3, 4]. Both the degree of obesity (i.e., body mass index (BMI) ≥ 30 kg/m^2^) and the length of time an individual has been obese influence their risk of CVD [5]. High-density lipoprotein cholesterol and low-density lipoprotein cholesterol are independent predictors of CVD [6–8].

Psychosocial factors are increasingly being recognized for their impact on CVD risk. Such factors include depression, social support, and ethnic identity. It is well documented that individuals with severe depression are at increased risk of developing a CVD and have higher CVD mortality rates than the general population [9, 10]. Some studies indicate that high levels of social support lead to longevity, better prognosis in depressed cardiac patients, successful recovery from a cardiac event, and efficacious coping methods [11–13]. A 13-year longitudinal study found that depression increased the risk for coronary heart disease among persons with low social support whereas it was not related to coronary heart disease risk in those with high levels of social support [14]. Authors of a 10-year literature review found that studies suggest depressive symptoms and a lack of social support are risk factors, potentially independent of each other, for adverse outcome in patients with heart disease [15]. Additionally, some evidence suggests that depressive symptoms and social support are independent predictors of adverse outcomes [15]. Acculturation has also been linked to CVD conditions. A study among adult Native Hawaiians found an association between having a stronger identification with the American culture/lifestyle and having hypertension when compared with those who do not, suggesting that acculturative factors may play a role in CVD risk [16].

Like many chronic diseases, the burden of CVD and its associated risk factors are not equally distributed across racial and ethnic groups. Despite Hawai‘i’s reputation as one of the healthiest states in the US, over one-third of all deaths in Hawai‘i are attributable to CVD [17, 18]. Data from the 2017 Hawai‘i Behavioral Risk Factor Surveillance Survey suggests difference in prevalence of hypertension, 31% in Japanese, 31% in Filipinos, 33% in Native Hawaiians, and 22% in non-Hispanic Whites [19]. Data from the National Health Interview Survey shows that Native Hawaiians have a greater prevalence of heart disease relative to non-Hispanic Whites, non-Hispanic Blacks, and Asians [20]. Analysis of health care data in Hawai‘i indicates that Filipinos and Native Hawaiians have the highest prevalence of type 2 diabetes compared with Asians and non-Hispanic Whites across age groups [21]. However, Native Hawaiians have the highest rates of myocardial infarction, CVD, and stroke of any ethnic group and are 3 to 4 times more likely to be afflicted with CVD and stroke, relative to non-Hispanic Whites [19, 21, 22].

One measure of CVD risk that takes specific behavioral and biological factors into account is the Framingham Risk Score (FRS). It estimates gender-specific, individual 10-year risk according to the level of exposure to different risk factors incorporated in a mathematical function [23, 24]. The FRS takes into account gender, smoking, total cholesterol, high-density lipoprotein cholesterol, age, and use of hypertension medication in determining CVD risk [24, 25]. The FRS is a point-based system that then turns the score into a percentage representing the likelihood of developing CVD in the next 10 years. For example, for females, a score of 14 translates to an 11.7% chance of developing CVD in the next 10 years. That same score translates to an 18.4% chance in males.

The purpose of this study is to examine differences in CVD risk across the four largest ethnic groups in Hawai‘i, after accounting for relevant sociodemographic, biological, and psychosocial factors that may explain differences. Understanding ethnic differences in CVD risk can aid in developing targeted prevention programs. No study to date has conducted cross-ethnic comparisons that included a diverse sample of disaggregated Asian and Pacific Islanders populations regarding CVD risk.

## Methods

Secondary data analysis of the Kohala Health Research (KHR) Project dataset is the basis for this study. KHR was a cross-sectional, community-based epidemiological study conducted between 1994 and 2001 in the North Kohala district on the island of Hawai‘i. Approximately 3000 people were invited to participate. This represented all non-pregnant adults (≥ 18 years of age) who resided in the community of interest during the study period. A total of 1462 participants completed the entire clinical examination and surveys. All participants provided written informed consent prior to participating in research activities. The University of Hawai‘i Committee on Human Subjects approved the protocol.

KHR procedures have been described in detail in previous publications [26, 27]. Briefly, the KHR Project had a two-prong recruitment approach. Native Hawaiians were identified via a database from a previous epidemiological study of Native Hawaiians only. Other participants (both Native Hawaiian and non-Native Hawaiian) were recruited via telephone, local public television announcements, flyers posted in local stores and community centers, and presentations at community organizations. Participants underwent a 2-h clinical examination and interview-administered assessments at KHR’s community-based clinic. The assessments consisted of sociodemographic, medical, psychosocial, behavioral, and sociocultural measures.

Due to the cross-sectional nature of our data, actual CVD events are not observable. FRS was chosen as a CVD risk estimate because it has been validated in multiple ethnic groups. In Hispanic, non-Hispanic Black, and non-Hispanic White cohorts, FRS predicted atrial fibrillation well [28]. Other studies have found that the risk factors used to calculate FRS predict CVD mortality equally well in non-Hispanic Blacks, Hispanics, and non-Hispanic Whites [29]. A study in the Malaysian population found that FRS models were able to stratify cardiovascular risk [30]. FRS is clinically effective at identifying patients at risk and can help physicians to effectively manage patients’ risk [31, 32].

### Assessment Measures

#### Sociodemographics

A personal history data form that included gender, age, education level, marital status, and ethnic ancestry collected sociodemographic data. Ethnic ancestry data was based on the participants’ self-report of blood quantum (i.e., less than 25%, 25–49%, 50–74%, 75–99%, and 100%) [33, 34]. Participants were defined as Native Hawaiian if they reported having any Native Hawaiian blood quantum descent from individuals living in the Hawaiian Islands prior to Western contact in 1778. Participants were defined as non-Hispanic White if they reported only non-Hispanic White ancestry, Filipino if they reported only Filipino ancestry, and Japanese if they reported only Japanese ancestry. The ethnicity classification schema is considered standard in Hawai‘i [35]. Except for Native Hawaiians, individuals who reported multiple ethnic ancestries were excluded from this analysis due to small numbers.

#### Clinical and Medical History

The KHR Project designed and used a detailed clinical and medical history form to collect self-report health and medical data. These data included hypertension prescription medication use and personal and family medical history (e.g., hypertension, diabetes, heart disease). The clinical examinations included systolic and diastolic blood pressure, total cholesterol, high-density lipoprotein cholesterol, and anthropometric measurements (i.e., weight, height) collected according to standardized protocols [26]. The FRS was used to estimate an individual’s CVD risk. Participants’ age, gender, smoking status, total cholesterol, high-density lipoprotein, and systolic blood pressure were entered into an online FRS calculator provided by the National Institutes of Health to obtain gender-specific 10-year CVD risk scores. Overall, lower scores indicate lower 10-year CVD risk but correspond to different percentage of risk for each gender [25]. FRS for males range from ≤ − 3 to > 18 while scores for females range from ≤ − 2 to > 21. Both the male and female 10-year CVD risk percentage range correspond to < 1% to > 30%. Thus, males have higher 10-year risk at lower FRS relative to females.

#### Depressive Symptoms

The 20-item Center for Epidemiological Studies-Depression (CES-D) Scale was used to assess frequency of depressive symptoms [36]. The CES-D has four subscales that assess four domains. Negative affect includes feelings of loneliness and sadness; lack of positive affect includes the absence of feelings of happiness and enjoyment; somatic complaints include physical symptoms; and interpersonal problems include symptoms related to interpersonal interactions. It measures the frequency with which participants have experienced a specific symptom within the previous week, using a 4-point rating scale that ranged from 0 = “rarely to none of the time” to 3 = “most or all of the time”. CES-D scores range from 0 to 60, where higher scores indicate greater frequency of depressive symptoms.

#### Social Support

A 6-item modified scale from the 10-item Lubben Social Network Scale (LSNS) was used to measure social support [37]. Participants were asked to rate the assistance and support they received from family and friends for diverse needs, ranging on a scale of 1 = “definitely true” to 5 = “definitely false.” The scoring ranges from 6 to 30, where lower scores indicate greater social support.

#### Cultural Identity

Cultural identity was measured using an 8-item scale comprised of two subscales, Ethno-Cultural Identity Subscale (ECIS) and American Cultural Identity Subscale (ACIS) [16, 38]. The subscales assessed participants’ knowledge of, involvement with, positive feeling toward, and perceived importance of their own ethno-cultural and American cultural backgrounds, respectively. Each subscale consists of 4 items scored on a 5-point Likert scale from 5 (very knowledgeable, very positive, very important) to 1 (not knowledgeable, not involved, and not important). Total subscale scores range from 4 to 20; higher scores indicate a stronger affiliation with their specific ethnic group’s culture or the American culture as indicated by the specific subscale.

### Data Analysis

Participant characteristics were summarized by mean (*M*) and standard deviation (SD) for continuous variables and frequencies and percentages for categorical variables. Participants who had a history of heart disease were excluded from this analysis. Differences in participant characteristics across the four race/ethnicity groups were examined using chi-squared or Fisher’s exact tests for categorical variables. Fisher’s exact tests were performed when the expected number of frequencies in a cell was less than 5, in which case chi-squared test would not have been valid and one-way ANOVA or Kruskal-Wallis tests for continuous variables. The Kruskal-Wallis test was used rather than ANOVA when the assumption of normality was violated. As a follow-up to the significant ANOVA or Kruskal-Wallis tests, post hoc analysis was performed to confirm where the significant differences occurred between groups. Pearson and Spearman rank correlation coefficients were calculated to examine the bivariate relationships between variables. For non-normal continuous variables, Spearman correlations were used rather than Pearson correlations. Multiple linear regressions for each sex on FRS were then conducted with the variables that showed significant bivariate association with FRS and were hierarchically assessed by adjusted R-square to select the best regression model. R-square changes were evaluated by the *F* test. Model 1 included ethnicity only. Model 2 added sociodemographic variables (i.e., age, education, and marital status). Model 3 added biological and psychosocial variables (i.e., BMI, CES-D, and ECIS). Least square means of FRS with standard errors were calculated for the four ethnic groups by the 10-year age group, adjusting for the covariate in model 3, above. In post hoc analysis, interaction terms between variables were also investigated. All data analyses were performed in SAS 9.4 (Cary, NC, 2011) and a two-tailed *p* value of less than 0.05 was regarded as statistically significant.

## Results

### Participant Characteristics

Data from 1146 individuals were included in these analyses. Table 1 summarizes participant’s characteristics. Overall, participants had a mean age of 48.8 ± 15.3 years; a mean FRS of 13.9 ± 6.9; and 55% were female. The median age was 47 years for the total sample, 47.5 for males and 47 for females. Native Hawaiians comprised the largest ethnic group (44.4%), followed by non-Hispanic Whites (25%), Filipinos (15.4%), and Japanese (15.3%). The majority (64.6%) had a high school degree or less and 75.4% had an annual household income below $50,000.

**Table 1:** Participant characteristics

Characteristics	Native Hawaiians, *N* = 509	Filipinos, *N* = 176	Japanese, *N* = 175	Whites, *N* = 286	Combined, *N* = 1146
Gender					
Male	223 (43.9)	65 (36.9)	83 (47.4)	140 (49.0)	511 (44.6)
Female	285 (56.1)	111 (63.1)	92 (52.6)	146 (51.1)	634 (55.4)
Age***	44.2 ± 14.4 _a_	52.8 ± 16.0 _a_	57.8 ± 16.3 _a_	48.0 ± 13.9 _a_	48.8 ± 15.3
Education level***					
Less than high school	48 (10.0) _f_	62 (35.2) _a_	21 (12.0) _f_	7 (2.5) _f_	138 (12.3)
High school graduate/GED	327 (67.8) _a_	62 (35.2) _n,w_	85 (48.6) _n_	111 (38.8) _a_	585 (52.3)
Some college	82 (17.0)	31 (17.6)	37 (21.1)	54 (18.9)	204 (18.2)
College graduate	25 (5.2) _a_	21 (11.9) _n_	32 (18.3) _n_	114 (39.9) _n_	192 (17.2)
Marital status***					
Never married	113 (22.3) _f,w_	21 (12.0) _n_	24 (13.7)	49 (17.1) _n_	207 (18.1)
Currently married	315 (62.3)	121 (69.1)	123 (70.3)	170 (59.4)	729 (63.8)
Separated	10 (2.0)	2 (1.1)	2 (1.1)	4 (1.4)	18 (1.6)
Widowed	34 (6.7)	22 (12.6) _w_	19 (10.9)	9 (3.2) _f_	84 (7.4)
Divorced	34 (6.7)	9 (5.1)	7 (4.0)	54 (18.9)	104 (9.1)
Annual household income***					
< $15,000	60 (12.5) _f,w_	38 (21.6) _n_	32 (18.4)	51 (18.0) _n_	181 (16.2)
$15,000–$24,999	87 (18.1)	41 (23.3)	43 (24.7)	48 (16.9)	219 (19.6)
$25,000–$34,999	116 (24.1)	45 (25.6)	28 (16.1)	41 (14.4)	230 (20.6)
$35,000–$49,999	99 (20.6)	24 (13.6)	26 (14.9)	62 (21.8)	211 (18.9)
$50,000–$74,999	97 (20.2)	23 (13.1)	29 (16.7)	46 (16.2)	195 (17.5)
> $74,999	22 (4.6)	5 (2.8)	16 (9.2)	36 (12.7)	79 (7.1)
Framingham Risk Score***					
Male	13.7 ± 7.3 _j_	15.0 ± 7.7	17.4 ± 5.8 _n,w_	14.2 ± 5.7 _j_	14.6 ± 6.8
Female	12.3 ± 7.5 _f,j_	15.6 ± 6.5 _n,w_	16.7 ± 6.0 _n,w_	11.8 ± 5.7 _f,j_	13.4 ± 7.0
Hypertension medication?***					
No	32 (27.8)	14 (26.9)	9 (14.5) _w_	23 (67.7) _j_	78 (29.7)
Yes	82 (71.3)	38 (73.1)	53 (85.5)	11 (32.4)	184 (70.0)
Don’t know	1 (0.9)	0 (0.0)	0 (0.0)	0 (0.0)	1 (0.4)
Diabetes status					
No	415 (81.7) _w_	144 (81.8) _w_	141 (80.6) _w_	275 (96.2) _n,f,j_	975 (85.2)
Yes	93 (18.3)	32 (18.2)	34 (19.4)	11 (3.8)	170 (14.8)
CES-D score***	10.4 ± 7.4 _j,w_	10.4 ± 7.8 _w_	8.5 ± 6.9 _n_	8.4 ± 8.0 _n,f_	9.6 ± 7.6
LSNS score***	8.9 ± 3.6 _w_	9.6 ± 3.6	9.3 ± 3.3	10.1 ± 4.5 _n_	9.4 ± 3.8
ACS***	10.2 ± 2.7 _j,w_	9.8 ± 3.1 _j,w_	8.9 ± 2.6 _n,f_	8.9 ± 2.9 _n,f_	9.6 ± 2.8
ECS***	8.1 ± 2.5 _f,w_	6.8 ± 2.4 _a_	7.8 ± 2.3 _f,w_	8.7 ± 2.8 _a_	8.0 ± 2.6

*CES-D*, Center for Epidemiological Studies-Depression; *LSNS*, Lubben Social Network Scale; *ACS*, American Cultural Subscale; *ECS*, Ethno-Cultural Subscale. *p < 0.05; **p < 0.01; ***p < 0.001, a: all pairwise comparisons, n: vs. Native Hawaiian, f: vs. Filipino, j: vs. Japanese w: vs. White

There were significant differences across the ethnic groups on many of the variables assessed including age, education, marital status, income, hypertension medication, FRS, depression, social support, American cultural identity, and ethno-cultural identity. Among males, mean FRS for Japanese was significantly higher than mean FRS for non-Hispanic Whites and Native Hawaiians. Among females, mean FRS for Japanese and Filipinos were significantly higher than those for non-Hispanic Whites and Native Hawaiians. Both Filipinos and Native Hawaiians had significantly higher depression scores than non-Hispanic Whites. Non-Hispanic Whites had significantly higher social support scores than Native Hawaiians. American cultural identity scores were significantly higher among non-Hispanic Whites and Japanese compared with Native Hawaiians and Filipinos. Finally, Filipinos had the lowest ethno-cultural identity scores while non-Hispanic Whites had the highest.

### Bivariate Correlations

Pearson correlation coefficients were calculated to examine the bivariate correlations between variables. An intercorrelation matrix is presented in Table 2. Women had lower FRS than men (*r* = − 0.09, *p* < 0.01). Higher FRS was associated with lower ethno-cultural identity (*r* = − 0.07, *p* < 0.04) and fewer depressive symptoms (*r* = − 0.09, *p* < 0.01). Age (*r* = 0.74, *p* < 0.001) and use of hypertension medication (*r* = 0.44, *p* < 0.001) were both positively associated with FRS, indicating that older participants and who used hypertension medication were likely to have higher FRS.

**Table 2:** Summary of bivariate correlations, means, and standard deviations

	Age	Edu	Income	HTN Rx	Diabetes	CES-D	LSNS	ECS	ACS	FRS
Gender	− 0.01	− 0.07*	− 0.03	0.06	0.01	0.04	− 0.12***	− 0.04	− 0.02	− 0.09**
Age	–	− 0.14***	− 0.18***	0.31***	0.23***	− 0.08**	− 0.04	− 0.11***	− 0.07*	0.74***
Education		–	0.29***	− 0.06	− 0.14***	− 0.13***	0.11***	− 0.00	− 0.23***	− 0.06*
Income			–	− 0.09	− 0.05	− 0.11***	− 0.03	0.07*	− 0.011***	− 0.05
Hypertension medication				–	0.14	− 0.02	− 0.11	− 0.09	0.08	0.44***
Diabetes status					–	0.04	− 0.05	− 0.07*	0.07*	0.28***
CES-D						–	0.33***	0.13***	0.15***	− 0.09**
LSNS							–	0.19***	0.11***	− 0.04
ECS								–	0.43***	− 0.07*
ACS									–	− 0.03
FRS										–
*M*						9.6	9.4	8.0	9.6	13.9
SD						7.6	3.8	2.6	2.8	6.9

*CES-D*, Center for Epidemiological Studies-Depression; *LSNS*, Lubben Social Network Scale; *ECS*, Ethno-Cultural Subscale; *FRS*, Framingham Risk Score; *HTN Rx*, hypertension medication. *p < 0.05; **p < 0.01; ***p < 0.001

### Hierarchical Multiple Regression

Hierarchical multiple regression was used to identify the best model to predict FRS for males and females separately (Tables 3 and 4, respectively). Only variables found to have a significant bivariate association with ethnicity and FRS were entered into the modeling for parsimony. Among both genders, model 1 only included ethnicity; model 2 included age, education, diabetes status, and marital status (i.e., sociodemographics); and model 3 added BMI, depression, and ethno-cultural identity. Among males, model 2 accounted for significantly more variance in FRS than model 1 (*R*^2^ = 0.56, *F* = 390.39, *p* < 0.001). Model 2 showed that Native Hawaiians (*B* = 1.46, *p* = 0.02) had FRS that were significantly higher than those in non-Hispanic Whites, controlling for age, education, and marital status. Model 3 accounts for significantly more variance in FRS (*R*^2^ = 0.59, *F* = 24.09, *p* < 0.001); however, ethnic differences in FRS are non-significant.

**Table 3:** Hierarchical regression analysis of ethnicity, age, education, and BMI on Framingham Risk Scores for males

Variables	Ethnicity								
	Model 1			Model 2			Model 3		
	*B*	SE *B*	*p* value	*B*	SE *B*	*p* value	*B*	SE *B*	*p* value
Hawaiian vs. White	− 0.54	0.77	0.48	1.50	0.60	0.02	0.79	0.61	0.20
Filipino vs. White	0.73	1.08	0.50	0.28	0.78	0.72	0.45	0.77	0.56
Japanese vs. White	3.18	1.00	0.002	0.82	0.71	0.25	1.04	0.69	0.13
Age				0.33	0.02	< 0.001	0.33	0.02	< 0.001
Education				0.59	0.27	0.03	0.57	0.26	0.03
Single vs. interrupt marital status				− 0.15	0.80	0.85	− 0.38	0.79	0.63
Married vs. interrupted marital status				1.10	0.63	0.07	1.07	0.62	0.09
Diabetes status				2.25	0.68	0.001	1.79	0.66	0.01
BMI							0.20	0.04	< 0.001
CES-D							− 0.06	0.03	0.03
ECS							0.10	0.08	0.22
*R*^2^	0.04			0.56			0.59		
*F* for change in *R*^2^				390.39 (*p* < 0.001)			24.09 (*p* < 0.001)

**Table 4:** Hierarchical regression analysis of ethnicity, age, education, and BMI on Framingham Risk Scores for females

Variable	Model 1			Model 2			Model 3		
	*B*	SE *B*	*p* value	*B*	SE *B*	*p* value	*B*	SE *B*	*p* value
Hawaiian vs. White	0.47	0.71	0.51	1.67	0.52	0.001	0.62	0.52	0.24
Filipino vs. White	3.85	0.88	< 0.01	1.54	0.64	0.02	1.90	0.64	0.003
Japanese vs. White	4.88	0.94	< 0.01	1.30	0.66	0.05	1.48	0.63	0.02
Age				0.38	0.02	< 0.001	0.38	0.02	< 0.001
Education				0.32	0.24	0.18	0.57	0.23	0.01
Single vs. interrupt marital status				0.62	0.74	0.40	0.15	0.71	0.83
Married vs. interrupted marital status				0.64	0.50	0.20	0.41	0.48	0.40
Diabetes status				1.59	0.54	0.004	0.77	0.53	0.15
BMI							0.23	0.03	< 0.001
CES-D							− 0.01	0.02	0.79
ECS							0.07	0.08	0.34
*R*^2^	0.07			0.59			0.63		
*F* for change in *R*^2^				418.96 (*p* < 0.001)			24.09 (*p* < 0.001)		

Among females, model 2 showed that non-Hispanic Whites had significantly lower FRS relative to Native Hawaiians, Filipinos, and Japanese (*R*^2^ = 0.59, *F* = 418.96, *p* < 0.001), controlling for age, education, and marital status. In model 3, FRS for non-Hispanic Whites remained significantly lower than FRS for Japanese and Filipinos. Additionally, a greater portion of the variance in FRS was explained in model 3 (*R*^2^ = 0.63, *F* = 24.09, *p* < 0.001). Interestingly, among both males and females, education was significantly associated with FRS such that greater education was related to higher FRS (males *B* = 0.57, *p* = 0.03; females *B* = 0.57, *p* = 0.01).

### Least Square Means

Tables 5 and 6 present the least square means of FRS for males and females, respectively, controlling for education, marital status, diabetes status, BMI, depression, and cultural identity. Among males, the only significant differences in FRS were in the 50- to 59-year-old age group. In this age group, Native Hawaiians and Filipinos had significantly higher FRS than non-Hispanic Whites. Among females, Native Hawaiians had significantly higher FRS compared with non-Hispanic Whites in the 50- to 59-year-old age group. In the oldest age group, those over 70 years old, Filipinos and Japanese had significantly higher FRS than non-Hispanic Whites.

**Table 5:** General linear model estimates of least square means of Framingham Risk Score by ethnicity and age group, after adjusting for covariates for males

Variables	Ethnicity			
	Native Hawaiian, *N* = 223	Filipino, *N* = 65	Japanese, *N* = 83	Non-Hispanic White, *N* = 140
≤ 39 years	8.86 ± 1.28	8.55 ± 1.63	11.63 ± 2.09	10.08 ± 1.66
≥ 40 years to ≤ 49 years	13.45 ± 0.87	10.96 ± 2.69	14.08 ± 1.31	14.21 ± 0.93
≥ 50 years to ≤ 59 years	17.75 ± 0.68 _w_	19.43 ± 1.01 _w_	16.74 ± 1.00	14.91 ± 0.61 _n,f_
≥ 60 years to ≤ 69 years	17.80 ± 1.02	16.15 ± 1.38	17.54 ± 1.20	17.70 ± 1.31
≥ 70 years	22.95 ± 1.60	21.34 ± 1.62	22.40 ± 0.97	22.07 ± 2.24

Adjusted for education, diabetes status, marital status, BMI, depression, and cultural identity. Subscripts indicate significance at *p* < 0.05 n: vs. Native Hawaiian, f: vs. Filipino, j: vs. Japanese w: vs. non-Hispanic White

**Table 6:** General linear model estimates of least square means of Framingham Risk Score by ethnicity, after adjusting for covariates for females

	Ethnicity			
Variables	Native Hawaiian, *N* = 285	Filipino, *N* = 111	Japanese, *N* = 92	Non-Hispanic White, *N* = 146
≤ 39 years	5.06 ± 0.77	5.65 ± 1.47	7.25 ± 1.83	4.43 ± 1.15
≥ 40 years to ≤ 49 years	11.78 ± 0.67	14.52 ± 1.12	15.17 ± 1.12	12.695 ± 0.70
≥ 50 years to ≤ 59 years	17.69 ± 0.73 _w_	16.96 ± 0.92	15.47 ± 1.05	14.81 ± 0.78 _n_
≥ 60 years to ≤ 69 years	18.89 ± 0.92	20.69 ± 0.94	19.93 ± 1.03	17.43 ± 1.12
≥ 70 years	21.10 ± 1.18	21.34 ± 0.80 _w_	21.73 ± 0.64 _w_	16.93 ± 1.22 _f,j_

Adjusted for education, marital status, diabetes status, BMI, depression, and cultural identity. Subscripts indicate significance at *p* < 0.05 n: vs. Native Hawaiian, f: vs. Filipino, j: vs. Japanese w: vs. non-Hispanic White

## Discussion

The purpose of this research was to examine potential ethnic-by-gender differences in 10-year risk for CVD based on the FRS, and after controlling for relevant sociodemographic, biological, and psychological factors. Contrary to what we expected, we failed to find any differences in FRS among men across ethnic groups after adjusting for age, education, marital status, BMI, depression, and cultural identity. Among females, we found that Filipinos had higher adjusted FRS than non-Hispanic Whites. However, when examining FRS by age group, we found that in both sexes, Native Hawaiians aged 50 to 59 years old had significantly higher FRS than non-Hispanic Whites. This was also true for Filipino males in the same age group. Among females over 70 years old, Filipinos and Japanese had higher FRS than non-Hispanic Whites. While not significant, Native Hawaiians in this age group also had an FRS that was higher than non-Hispanic Whites. It is possible that the lack of significances is due to the sample size of Native Hawaiian females in this age group. This increased CVD risk in the 50- to 59-year-old age group is supported by a greater prevalence of CVD in Native Hawaiians and Filipinos, relative to non-Hispanic Whites. The discrepancy between our reported FRS in the other age groups and CVD rates reported in the literature is not unique.

While FRS is clinically effective, it is an imperfect measure [39]. For instance, guidelines from New Zealand suggest that Framingham Risk Scores may underestimate risk in high-risk groups (including Maori and other Pacific Islanders in New Zealand) [40]. Previous research has shown that CVD risk in Filipinos varies significantly by risk calculator [41]. The FRS has been found to underestimate fatal CVD risk estimates in individuals with type 2 diabetes [42]. A retrospective, observational study using claims data in Hawai‘i found that Native Hawaiians and Filipinos had higher rates of hypertension and diabetes, relative to non-Hispanic Whites [21]. Data from the 2017 Behavioral Risk Factor Surveillance Survey also showed that Native Hawaiians and Filipinos have higher diabetes prevalence than non-Hispanic Whites and Japanese [43]. FRS may underestimate CVD in Native Hawaiians and Filipinos due to their higher rates of diabetes relative to other ethnic groups.

Interestingly, greater education was related to greater risk of CVD in both genders even after adjusting for age and ethnicity, both of which are associated with education and CVD risk. A possible explanation may be acculturation differences between younger and older age groups. The older Filipino participants may have immigrated to Hawai‘i for plantation work. They may have maintained more traditional lifestyles including diets, gardening and other yard maintenance, and physical activity routines. These traditional lifestyles are often healthier than their Westernized counterparts [44]. The younger generations may have more formal education but have adopted a more Western lifestyle (i.e., sedentary, poor diet, increased alcohol and tobacco consumption) [45]. These results are supported by other studies that have found a positive association between CVD risk and immigrants’ length of residence in the US [46–48]. Length of residence in the US has been associated with coronary heart disease in Chinese and South Asian immigrants [49, 50]. Researchers have found positive associations between acculturation and hypertension in multiple ethnic and US immigrant populations [51–53]. The impact of the interaction between age and education on FRS was explored but found not to be significant.

Other studies with Filipinos in Hawai‘i have found a positive association between length of time in Hawai‘i, as a measure of acculturation, and BMI [54]. Studies including Filipino immigrants to the US continent have found length of time in the US to be positively related to the likelihood of having hypertension [55]. Research comparing between Japanese men in Japan and Hawai‘i found the prevalence of coronary artery calcification, a precursor to coronary heart disease, in Hawai‘i was three times greater than that in Japan [56]. This magnitude of difference remained after controlling for a variety of health behavior risk factors. Increased risk of diabetes has also been found in Japanese immigrants to the US [57]. Our study operationalized ethnic identity as a participant’s affinity toward, knowledge of, and affiliation with American and their ethnic culture. Thus, length of residence in the US may be related to our measure of ethnic identity but are conceptually different.

Understanding ethnic differences in CVD risk can aid in developing targeted and effective prevention programs. Our results suggest that Filipino females have elevated CVD risk, relative to non-Hispanic White females in Hawai‘i. Differences in CVD risk are potentially related to the acculturation. Programs that promote traditional diets, activities, and culture may be effective means for decreasing cardiovascular disease risk. Recently, Kaholokula et al. (2017) found a hypertension education program that included hula, the traditional dance of Hawai‘i, significantly improved participant blood pressure compared with a control group [58]. A qualitative study with these participants found that participating in hula increased participants’ connection to their culture and increased their commitment to healthy lifestyle choices [59].

Results from this study should be interpreted in context of its limitations. Due to the cross-sectional design, the results support associations between variables but causation cannot be inferred. Additionally, while Framingham risk factors have been found to equally predict CVD mortality in multiple ethnic groups, individual risk factors and CVD associations differ by race and ethnicity [29]. While all community residents were invited to participate in this study, just under half participated. Those who chose to participate may have systematically differed from those who did not participate in a way that is related to our variables of interest (i.e., age, education, depression, etc.). This self-selection bias may limit the generalizability of our results.

## Conclusion

Our study suggests that biological risk factors, such as BMI and age, and psychosocial risk factors, such as depression, explain a significant proportion of the variation in CVD risk as measured by FRS. Our results suggest that in males there is no difference in 10-year CVD risk between ethnic groups. Among females, Filipinos have a higher 10-year CVD risk compared with non-Hispanic Whites. However, data on CVD events indicate that Native Hawaiians of both sexes are at greater risk for CVD compared with other ethnic groups. Together, this provides evidence for the need to recalibrate FRS for Native Hawaiian and Filipino populations. Additionally, we need to better understand the factors that contribute to unexplained variance in CVD risk and address these factors in cardiovascular health promotion efforts.

## Data Availability

The dataset analyzed during the current study are available from the corresponding author on reasonable request.

## Abbreviations

ACS: American Cultural Subscale
ANOVA: Analysis of variance
BMI: Body mass index
CES-D: Center for Epidemiological Studies-Depression Scale
CHD: Coronary heart disease
CVD: Cardiovascular disease
ECS: Ethno-Cultural Subscale
FRS: Framingham Risk Score
KHR: Kohala Health Research
LSNS: Lubben Social Network Scale

## References

[1] Benjamin EJ, Virani SS, Callaway CW, Chamberlain AM, Chang AR, Cheng S, Chiuve SE, Cushman M, Delling FN, Deo R, de Ferranti SD, Ferguson JF, Fornage M, Gillespie C, Isasi CR, Jiménez MC, Jordan LC, Judd SE, Lackland D, Lichtman JH, Lisabeth L, Liu S, Longenecker CT, Lutsey PL, Mackey JS, Matchar DB, Matsushita K, Mussolino ME, Nasir K, O’Flaherty M, Palaniappan LP, Pandey A, Pandey DK, Reeves MJ, Ritchey MD, Rodriguez CJ, Roth GA, Rosamond WD, Sampson UKA, Satou GM, Shah SH, Spartano NL, Tirschwell DL, Tsao CW, Voeks JH, Willey JZ, Wilkins JT, Wu JH, Alger HM, Wong SS, Muntner P, American Heart Association Council on Epidemiology and Prevention Statistics Committee and Stroke Statistics Subcommittee (2018). Heart disease and stroke statistics 2018 update: a report from the American Heart Association. Circulation.

[2] Nelson S et al. Projections of cardiovascular disease prevalence and costs*.* 2016.

[3] National Heart Lung and Blood Institute, Know the differences: cardiovascular disease, heart disease, coronary heart disease. 2016, National Institutes of Health: https://www.nhlbi.nih.gov/sites/default/files/media/docs/Fact_Sheet_Know_Diff_Design.508_pdf.pdf. Accessed 30 Sept 2019.

[4] National High Blood Pressure Education Program, The seventh report of the Joint National Committee on Prevention, Detection, Evaluation, and Treatment of High Blood Pressure. 2004, National Institutes of Health: Bethesda.

[5] Ortega FB, Lavie CJ, Blair SN (2016). Obesity and cardiovascular disease. Circ Res.

[6] Boden WE (2000). High-density lipoprotein cholesterol as an independent risk factor in cardiovascular disease: assessing the data from Framingham to the Veterans affairs high-density lipoprotein intervention trial. Am J Cardiol.

[7] Gordon T, Castelli WP, Hjortland MC, Kannel WB, Dawber TR (1977). High density lipoprotein as a protective factor against coronary heart disease: the Framingham Study. Am J Med.

[8] Cromwell WC, Otvos JD, Keyes MJ, Pencina MJ, Sullivan L, Vasan RS, Wilson PWF, D’Agostino RB (2007). LDL particle number and risk of future cardiovascular disease in the Framingham Offspring Study—implications for LDL management. J Clin Lipidol.

[9] Goldston K, Baillie AJ (2008). Depression and coronary heart disease: a review of the epidemiological evidence, explanatory mechanisms and management approaches. Clin Psychol Rev.

[10] Hare DL, Toukhsati SR, Johansson P, Jaarsma T (2013). Depression and cardiovascular disease: a clinical review. Eur Heart J.

[11] Greco A, Steca P, Pozzi R, Monzani D, D’Addario M, Villani A, Rella V, Giglio A, Malfatto G, Parati G (2014). Predicting depression from illness severity in cardiovascular disease patients: self-efficacy beliefs, illness perception, and perceived social support as mediators. Int J Behav Med.

[12] Schwarzer R, K.N., Social support, in Health psychology, K.A. French D, Vedhara K, Weinman J, Editor. 2010, Wiley-Blackwell: Oxford. p. 283–293.

[13] Brummett BH, Babyak MA, Barefoot JC, Bosworth HB, Clapp-Channing NE, Siegler IC, Williams RB, Mark DB (1998). Social support and hostility as predictors of depressive symptoms in cardiac patients one month after hospitalization: a prospective study. Psychosom Med.

[14] Liu RT, Hernandez EM, Trout ZM, Kleiman EM, Bozzay ML (2017). Depression, social support, and long-term risk for coronary heart disease in a 13-year longitudinal epidemiological study. Psychiatry Res.

[15] Compare A (2013). Social support, depression, and heart disease: a ten year literature review. Front Psychol.

[16] Kaholokula JK, Iwane MK, Nacapoy AH (2010). Effects of perceived racism and acculturation on hypertension in Native Hawaiians. Hawaii Med J.

[17] National Center for Health Statistics. Stats of the state of Hawaii (2018). [cited 2019 June 28]; Available from: https://www.cdc.gov/nchs/pressroom/states/hawaii/hawaii.htm. Accessed 28 June 2019.

[18] Krisberg K. Hawaii again take lead spot as healthiest state in US rankings. The Nation’s Health, 2019. February/March(49 (1)): p. 11.

[19] Hawaii State Department of Health, The Hawaii Behavioral Risk Factor Surveillance System. 2017, Hawaii Health Data Warehouse, Indicator-Based Information System for Public Health: http://ibis.hhdw.org/ibisph-view/. Accessed 15 Aug 2019.

[20] Schiller J (2012). *Summary health statistics for U.S. adults:* National Health Interview Survey*,* 2010. Vital Health Stat Rep.

[21] Juarez DT, Davis JW, Brady SK, Chung RS (2012). Prevalence of heart disease and its risk factors related to age in Asians, Pacific Islanders, and Whites in Hawai’i. J Health Care Poor Underserved.

[22] Nakagawa K, Koenig MA, Asai SM, Chang CW, Seto TB (2013). Disparities among Asians and native Hawaiians and Pacific Islanders with ischemic stroke. Neurology.

[23] O’Donnell CJ, Elosua R (2008). *Cardiovascular risk factors. Insights from Framingham Heart study*. Revista Espanola de Cardiologia (English Edition).

[24] D’Agostino RB (2008). General cardiovascular risk profile for use in primary care. Circulation.

[25] D’Agostino RB, Grundy S, Sullivan LM, Wilson P, for the CHD Risk Prediction Group (2001). Validation of the Framingham coronary heart disease prediction scores: results of a multiple ethnic groups investigation. Jama.

[26] Grandinetti A, Chang HK, Mau MK, Curb JD, Kinney EK, Sagum R, Arakaki RF (1998). Prevalence of glucose intolerance among Native Hawaiians in two rural communities. Native Hawaiian Health Research (NHHR) Project. Diabetes Care.

[27] Mau MK, Grandinetti A, Arakaki RF, Chang HK, Kinney EK, Curb JD, Native Hawaiian Health Research (NHHR) Project (1997). The insulin resistance syndrome in native Hawaiians. Native Hawaiian Health Research (NHHR) Project. Diabetes Care.

[28] Shulman E, Kargoli F, Aagaard P, Hoch E, di Biase L, Fisher J, Gross J, Kim S, Krumerman A, Ferrick KJ (2016). Validation of the Framingham Heart Study and CHARGE-AF risk scores for atrial fibrillation in Hispanics, African-Americans, and non-Hispanic Whites. Am J Cardiol.

[29] Hurley LP, Dickinson LM, Estacio RO, Steiner JF, Havranek EP (2010). Prediction of cardiovascular death in racial/ethnic minorities using Framingham risk factors. Circ Cardiovasc Qual Outcomes.

[30] Selvarajah S, Kaur G, Haniff J, Cheong KC, Hiong TG, van der Graaf Y, Bots ML (2014). Comparison of the Framingham Risk Score, SCORE and WHO/ISH cardiovascular risk prediction models in an Asian population. Int J Cardiol.

[31] Jacobson TA, Gutkin SW, Harper CR (2006). Effects of a global risk educational tool on primary coronary prevention: the Atherosclerosis Assessment Via Total Risk (AVIATOR) study. Curr Med Res Opin.

[32] Wells S, Furness S, Rafter N, Horn E, Whittaker R, Stewart A, Moodabe K, Roseman P, Selak V, Bramley D, Jackson R (2008). Integrated electronic decision support increases cardiovascular disease risk assessment four fold in routine primary care practice. Eur J Cardiovasc Prev Rehabil.

[33] Kaholokula JKA (2006). Ethnic-by-gender differences in cigarette smoking among Asian and Pacific Islanders. Nicotine Tob Res.

[34] Kaholokula JKA (2003). Biological, psychosocial, and sociodemographic variables associated with depressive symptoms in persons with type 2 diabetes. J Behav Med.

[35] Braun KL, Look MA, Yang H, Onaka AT, Horiuchi BY (1996). Native Hawaiian mortality, 1980 and 1990. Am J Public Health.

[36] Radloff LS (1977). The CES-D scale: a self-report depression scale for research in the general population. Appl Psychol Meas.

[37] Lubben JE (1988). Assessing social networks among elderly populations. Family Commun Health.

[38] Kaholokula JK, Nacapoy AH, Grandinetti A, Chang HK (2008). Association between acculturation modes and type 2 diabetes among Native Hawaiians. Diabetes Care.

[39] Damen JA, Pajouheshnia R, Heus P, Moons KGM, Reitsma JB, Scholten RJPM, Hooft L, Debray TPA (2019). Performance of the Framingham risk models and pooled cohort equations for predicting 10-year risk of cardiovascular disease: a systematic review and meta-analysis. BMC Med.

[40] New Zealand Guideline Group, The assessment and management of cardiovascular risk. 2003: Wellington, New Zealand.

[41] Ancheta IB, Battie CA, Volgman AS, Ancheta CV, Palaniappan L (2017). Cardiovascular disease risk score: results from the Filipino–American Women Cardiovascular Study. J Racial Ethn Health Disparities.

[42] Coleman RL, Stevens RJ, Retnakaran R, Holman RR (2007). Framingham, SCORE, and DECODE risk equations do not provide reliable cardiovascular risk estimates in type 2 diabetes. Diabetes Care.

[43] Diabetes - prevalence, age adjusted, Hawaii Health Data Warehouse, Hawaii State Department of Health, Behavioral Risk Factor Surveillance System, 2017.

[44] Jin K, Gullick J, Neubeck L, Koo F, Ding D (2017). Acculturation is associated with higher prevalence of cardiovascular disease risk-factors among Chinese immigrants in Australia: evidence from a large population-based cohort. Eur J Prev Cardiol.

[45] Warehouse HHD. Education level by age group, in Behavioral Risk Factor Surveillance System. 2011, Hawaii State Department of health: Honolulu.

[46] Daviglus ML, Talavera GA, Avilés-Santa ML, Allison M, Cai J, Criqui MH, Gellman M, Giachello AL, Gouskova N, Kaplan RC, LaVange L, Penedo F, Perreira K, Pirzada A, Schneiderman N, Wassertheil-Smoller S, Sorlie PD, Stamler J (2012). Prevalence of major cardiovascular risk factors and cardiovascular diseases among Hispanic/Latino individuals of diverse backgrounds in the United States. Jama.

[47] Le-Scherban F (2016). Immigrant status and cardiovascular risk over time: results from the multi-ethnic study of atherosclerosis. Ann Epidemiol.

[48] Commodore-Mensah Y, et al. Length of residence in the United States is associated with a higher prevalence of cardiometabolic risk factors in immigrants: a contemporary analysis of the National Health Interview Survey. J Am Heart Assoc. 2016;5(11). 10.1161/JAHA.116.004059 PMC5210341 27815269

[49] Gong Z, Zhao D (2016). Cardiovascular diseases and risk factors among Chinese immigrants. Intern Emerg Med.

[50] Dodani S, Dong L (2011). Acculturation, coronary artery disease and carotid intima media thickness in South Asian immigrants--unique population with increased risk. Ethn Dis.

[51] Moran A (2007). Acculturation is associated with hypertension in a multiethnic sample. Am J Hypertens.

[52] Teppala S, Shankar A, Ducatman A (2010). The association between acculturation and hypertension in a multiethnic sample of US adults. J Am Soc Hypertens.

[53] Divney AA, Echeverria SE, Thorpe LE, Trinh-Shevrin C, Islam NS (2019). Hypertension prevalence jointly influenced by acculturation and gender in US immigrant groups. Am J Hypertens.

[54] Novotny R, Williams AE, Vinoya AC, Oshiro CES, Vogt TM (2009). US acculturation, food intake, and obesity among Asian-Pacific hotel workers. J Am Diet Assoc.

[55] Ursua RA, Islam NS, Aguilar DE, Wyatt LC, Tandon SD, Abesamis-Mendoza N, Nur PRMQ, Rago-Adia J, Ileto B, Rey MJ, Trinh-Shevrin C (2013). Predictors of hypertension among Filipino immigrants in the Northeast US. J Community Health.

[56] Abbott RD, Ueshima H, Rodriguez BL, Kadowaki T, Masaki KH, Willcox BJ, Sekikawa A, Kuller LH, Edmundowicz D, Shin C, Kashiwagi A, Nakamura Y, el-Saed A, Okamura T, White R, Curb JD (2007). Coronary artery calcification in Japanese men in Japan and Hawaii. Am J Epidemiol.

[57] Salant T, Lauderdale DS (2003). Measuring culture: a critical review of acculturation and health in Asian immigrant populations. Soc Sci Med.

[58] Kaholokula JKA (2017). Cultural dance program improves hypertension management for Native Hawaiians and Pacific Islanders: a pilot randomized trial. J Racial Ethn Health Disparities.

[59] Maskarinec GG, Look M, Tolentino K, Trask-Batti M, Seto T, de Silva M, Kaholokula JK‘ (2015). Patient perspectives on the Hula Empowering lifestyle adaptation study: benefits of dancing hula for cardiac rehabilitation. Health Promot Pract.

